# CHARACTERISTICS OF DUALLY ELIGIBLE BENEFICIARIES WHO RECEIVE MEDICARE HOME HEALTH CARE SERVICES: A SCOPING REVIEW

**DOI:** 10.1093/geroni/igae098.1423

**Published:** 2024-12-31

**Authors:** Ayomide Bankole, Dorothy Addo-Mensah, Jamie Conklin

**Affiliations:** Duke University, Durham, North Carolina, United States; University of North Carolina, Chapel Hill, North Carolina, United States; University of North Carolina, Chapel Hill, North Carolina, United States

## Abstract

Medicare Home Healthcare (HHC) is a commonly utilized post-acute care service by Medicare beneficiaries, including those with dual Medicare and Medicaid coverage (dual eligibles). Dual eligibles experience inequities in HHC as they report poorer health outcomes (e.g re-hospitalizations) than other Medicare beneficiaries in HHC. Dual eligibles may also experience challenges navigating and coordinating care across two payers of healthcare (Medicare and Medicaid). To improve health outcomes of dual eligible beneficiaries within HHC, there is a need to understand characteristics of this population and components of successful interventions within this setting. Thus, we conducted a scoping review to examine available evidence on dual eligibles who receive Medicare HHC. Research questions: (1) What are the characteristics of dual eligibles who receive HHC? (2) What health outcomes have been described and (3) which interventions influence these outcomes? With consultation from a Health Services Librarian, a search was conducted across four databases (PubMed, CINAHL, APA Psych Info, and Scopus) on January 10, 2024. After removing duplicates, 1,425-articles were identified for screening. Using Covidence software, reviewers worked in pairs to screen the articles. This phase yielded 83-articles. Given the changes in Medicaid eligibility and policies over the past decade, we then limited articles to those published within the last 10-years; leaving 53-articles for data extraction. We will extract and synthesize data using narrative-synthesis and descriptive-statistics, guided by the research questions. In this presentation, we will describe characteristics of dual eligibles receiving Medicare HHC and recommend strategies to improve their health outcomes within this setting.

